# Comparison of Aripiprazole and Risperidone effectiveness in treating obsessive‐compulsive disorder in patients with bipolar disorder: Double‐blind, randomized clinical trial

**DOI:** 10.1002/hsr2.1531

**Published:** 2023-08-27

**Authors:** Faezeh Khorshidian, Angela Hamidia, Farzan Kheirkhah, Ali Akbar Moghadamnia, Ali Bijani, Seyedeh Mahbobeh Mirtabar, Sakineh Javadian Koutanaei

**Affiliations:** ^1^ Department of Psychiatry, School of Medicine, Health Research Institute Babol University of Medical Sciences Babol Iran; ^2^ Department of Pharmacology, School of Medicine, Health Research Institute Babol University of Medical Sciences Babol Iran; ^3^ Department of Epidemiology, Social Determinants of Health Research Center, Health Research Institute Babol University of Medical Sciences Babol Iran; ^4^ Student Committee Research Babol University of Medical Sciences Babol Iran; ^5^ Clinical Research Development Unit of Rouhani Hospital Babol University of Medical Sciences Babol Iran

**Keywords:** antipsychotic agents, bipolar disorder, obsessive‐compulsive disorder, psychiatric comorbidity, treatment effectiveness

## Abstract

**Background:**

Obsessive‐compulsive disorder (OCD) is a mental illness with a chronic coarse and waxing and waning of symptoms. Treatment of OCD in patients with bipolar disorder (BD) remains challenging.

**Objectives:**

The present study aims to compare the safety and effectiveness of Risperidone and Aripiprazole as adjunctive therapy with valproate sodium, in treating mania, depression, and OCD in patients with comorbidity of OCD‐BD.

**Methods:**

This research is 3 phase, double‐blind, randomized clinical trial, with a total number of 64 patients. The diagnostic psychiatrist clinical interview was based on diagnostic and statistical manual of mental disorders, 5th edition (DSM‐5) criteria. For assessing severity of OCD, mania, and depression, Yale−Brown obsessive‐compulsive scale (Y‐BOCS), young mania rating scale (YMRS), and Hamilton depression rating scale (HAM‐D) scores were used. Patients were randomly assigned to the two parallel groups. All patients in both group were received valproate sodium, one group was treated with Aripiprazole and the other group was treated with Risperidon as adjective therapy with valproate sodium.

The SPSS software (version 22), *χ* ^2^ test, *t*‐test, and analysis of variance with repeated measures were used to analyze the data.

**Results:**

The dosage and time of both drugs were statistically significant in reducing the mean score of all three mentioned scales, but the effect of group was not statistically significant in HAM‐D and YMRS scores, only in terms of OCD, the mean of the Y‐BOCS score was significantly lower in the Aripiprazole group (*p* < 0.001). In relation to side effects, Risperidone induced statistically significant weight gain (*p* < 0.001) and Aripiprazole induced statistically significant sleep disturbance (*p* < 0.05).

**Conclusions:**

Both Aripiprazole and Risperidone can be used effectively as adjunctive therapy with valproate sodium in treating OCD in patients with BD without any serious and life threatening adverse effect. Aripiprazole is more effective than Risperidone in treating OCD in BD.

## Key points

- Adjunctive therapy of Aripiperazole or Risperidone to valproate sodium is effective in the treatment of obsessive‐compulsive disorder (OCD).
- Aripiperazole is more effective than risperidone in the treatment of OCD in bipolar patients.
- Higher dose and longer duration of treatment significantly increases the therapeutic effect of adjunctive therapy of these two drugs in the treatment of OCD in bipolar patients.

## 1 BACKGROUND

Obsessive‐compulsive disorder (OCD) is defined by repetitive unwanted intrusive thoughts, images, or urges (obsessions) that result in marked distress or anxiety in patients, and by recurrent behavioral or mental acts (compulsions) that the person feels driven to perform in response to an obsession or according to rules that must be applied rigidly to prevent event or situation or reducing distress.”^1^, ^2^ Studies showed its lifetime prevalence around 1%−2% worldwide, with slightly higher rate in females than males.^3^, ^4^, ^5^ Despite the low prevalence of OCD, it can have comorbid with many other psychiatric disorders with the prevalence up to 32%.^6^, ^7^ For instance, OCD and bipolar disorder (BD) are highly comorbid with each other.^8^, ^9^ Studies revealed lifetime outbreak rate of comorbid OCD in patients with BD, between 11.1% and 21%.^10^ Furthermore, in patients with BD, the risk of later diagnosis of OCD is about 1.2 times higher.^11^ OCD is more probably to be revealed during depressive episodes and mixed mania of BD,^12^, ^13^ and also OCD related to 2.4 times incensement in the probability of suicidal ideation among youngsters with BD.^14^ Comorbid of OCD‐BD has always been related to lower quality of life and poorer functioning than those with either BD or OCD alone.^15^ One of the most challenging problem in treating comorbidity of OCD‐BD is around the risk of selective serotonin‐reuptake‐inhibitors (SSRIs) in precipitating manic episode, because for the first line treatment of OCD without the history of BD, SSRI is the first choice.^16^ Also, there are limited data about the utility of cognitive‐behavioral therapy (CBT) in treating OCD in comorbid with BD.^17^ Furthermore, manic episodes are more frequent in patients with OCD‐BD who are not on mood stabilizers and sometimes we witnesses rapid cycling bipolar.^18^ Studies showed that treatment of manic episode and also major depressive episode in BD, with combination therapy of mood stabilizer and antipsychotic lead to faster onset of therapeutic effect, more significant remission, and greater therapeutic response than monotherapy of mood stabilizer or antipsychotic alone.^19^, ^20^, ^21^, ^22^, ^23^, ^24^, ^25^ Considering that OCD and BD are two separate disorder that can be seen as comorbidity in the same patient. Therefore, it is not the case that the treatment and resolution of one leads to the treatment of the other, and if the obsessive‐compulsive symptoms completely recovers after the treatment of BD, and if only during mood episodes, the patient experiences obsessive‐compulsive symptoms, in fact, these obsessive‐compulsive symptoms are caused by an increasing in the goal directed of the patient activity during his mood episode and is not considered OCD. Therefore, in the case of real comorbidity of these two disorders, independent treatment for OCD is required.^9^, ^10^

Therefore, due to the excistence of a challenge and risk of switch to mania in patients with OCD‐BD during treatment with SSRI and also due to the importance of the faster and greater therapeutic effect in treating mania and depression in BD and surplus the frequent use of Risperidone in psychiatric hospitals in Iran and with considering the documents beckon to the lower side effect profile of Aripiprazole, which causes less metabolic and hormonal problems and particularly despite the importance of these topics, there is limited and few research for comparison the safety and effectiveness of Risperidone and Aripiprazole in treating comorbid OCD‐BD in Iran and especially no study were done with similar pharmaceutical manufacturing company with due attention to the possible difference in influence of manufacturer's company according to the races (considering that our study was conducted on patients in the north of Iran) and also no study were done with clinical diagnostic interview by psychiatrist based on DSM‐5 (diagnostic and statistical manual of mental disorders, 5th edition) accompanied by intensification and determining the score with young mania rating scale (YMRS), Hamilton depression rating scale (HAM‐D), and Yale−Brown obsessive‐compulsive scale (Y‐BOCS). We aimed to design this randomized clinical trial to compare the effectiveness and side effects of Aripiprazole and Risperidone from Sobhan Darou Pharmaceutical Company for treating OCD, mania, and depression in patients with BD.

## 2 METHODS

### 2.1 Study design

This study is a single‐center, 3 phase, double blind, randomized clinical trial with parallel‐group that have been fully implented in the Yahya Nezhad Educational‐Medical Hospital of Babol University of Medical Sciences in Mazandaran. The informed consent forms, patient recruitment materials, and research protocol were investigated and certified by institutional ethics committees (IR.MUBABOL.REC.1399.072) and registered in the Iranian Registry of Clinical Trials (IRCT20200614047761N1).

### 2.2 Study population and sample size

The research were done in psychiatric inpatient wards and outpatient clinics of Yahya Nezhad Hospital in the year 2020. Inclusion criteria were participants with ages between 18 and 60 years old, with comorbid OCD‐BD, based on clinical diagnostic interview by psychiatrist on the basis of DSM‐5 criteria, that they had Y‐BOCS score ≥16 and HDRS score ≥17 and YMRS score >20, with absence of pregnancy or lactation in women, absence of psychotic disorder, absence of remarkable cognitive impairment or mental retardation, lack of physical illness that are contraindicated for taking Risperidone or Aripiprazole, and not being under psychotherapy during the study. The exclusion criteria were the severe new‐onset medical illness during the study, patients who didn't follow‐up for 4 weeks, and the occurrence of severe complications of drugs during the trial. The statistical specialist of the study assessed sample size at 64 patients to provide ≥80% power to diagnose, at least four units difference for Y‐BOCS score between groups. On entry, 64 patients were enrolled, they were assigned to each study group with a 1:1 ratio by random block allocation method by mean of an online tool at randomization.com website, the obtained sequence wrote in separate sheets and placed in closed envelopes by statistical specialist of research, other executors of the study didn't know the content of envelopes. Three patient didn't follow‐up and were excluded from research because they didn't want to continue drug treatment and take medicine. All these three patients were excluded in sixth weeks, one of them was from Risperidone group and two other were from Aripiprazole group, for blinding the pharmacologist who were adviser professor of research, first crushed drugs and then placed them in opaque capsules in same size and shape and coded them, codes were accessible only to pharmacologist of study and patients and other trial personnel remained blinded to them. Written informed consent was given by executor of study (psychiatry resident under supervision of psychiatry professors) to all patients or their legal representatives to take part in the research. The forms were based on local regulatory requirements and adhered to the ethical principles of the declaration of Helsinki. The executors received demographic information of all patients with preservation of confidential elements.

### 2.3 Interventions

In this research, the patients were divided into two equal groups. All patients were under treated of valproate sodium with dose between (1000 mg/day up to 20 mg/kg/day). One group of patients received the drug Aripiprazole along with valproate sodium and the other group received the drug Risperidone along with valproate sodium. The choice of drug dose was based on a meta‐analysis article that had investigated and reported the equivalent dose between Risperidone and Aripiprazole,^10^, ^26^, ^27^, ^28^ patients in the Aripiprazole group received Aripiprazole manufactured by Sobhan Darou Pharmaceutical Company with the primer dose of 2.5 mg/day, then slowly increased to 10 mg/day for the first 6 weeks and then 20 mg/day for the second 6 weeks. Patients in the Risperidone group received Risperidone manufactured by Sobhan Darou Pharmaceutical Company with the primer dose of 0.5 mg daily, then gradually increased to 2 mg/day for the first 6 weeks, and then 4 mg/day for the second 6 weeks (increasement of dose in both groups done for all participants). Clinical visits were scheduled for all participants at times 0, 6th, and 12th weeks of study to assess effectiveness, safety, and other outcomes of the interventions.

### 2.4 Measurements

We performed this article based on the Consort guidelines of reporting checklist for randomized trial. The diagnostic psychiatrist clinical interview was based on DSM‐5 criteria, according to it diagnosis of comorbid of OCD‐BD was made. For assessing severity of OCD, mania, and depression, Y‐BOCS, YMRS, and HAM‐D scores were used. Primary outcome was the treatment's effectiveness assessed by using the YMRS, HAM‐D, and Y‐BOCS scores at times 0, 6th, and 12th weeks of the intervention. The secondary outcome was treatment‐adverse events assessed at the 6th and 12th weeks of the intervention by using the questionnaire based on medscape website that prepared via project executor who were psychiatry professors. Y‐BOCS questionnaire is a semistructural interview designed to determine the OCD and its severity. Based on the research carried out by Esfahani et al.,^26^ the reliability and validity of its Persian version were 97% and 91%, respectively. On this scale, a score of fewer than 10 points shows very mild symptoms of OCD, a score of ≥16 points shows moderate symptoms, and a score of >25 shows severe symptoms. HAM‐D is designed to assess the symptoms of depression and its severity, it's range is from 0 to more than 23 points. On this scale, a score of 0−7 shows the absence of depression or remission, and a score of more than 23 shows severe depression. Based on the result of the study performed by Gharaei et al., its validity is high and it's reliability by means of test−retest method was 0.85 and 0.89.^29^ YMRS was designed to assess symptoms of mania, consists of 11 items, and it is semistructural questionnaire depends both on the patient's clinical condition in the last 48 h and also evaluator judgment, ranging from 0 to 60 points which score between 38 and 60 represent severe mania, and score ≤12 represent successful treatment. Based on the result of the study performed by Ebrahimi et al.,^30^ it had high homogeneity (*a* = 0.82) and validity (74.5%) among the Iranian population.

### 2.5 Statistical analysis

The sample size assessed at 64 patients to provide ≥80% power to diagnose, at least four units difference for Y‐BOCS score between groups. Variables were summarized using descriptive statistics such as mean and frequency based on the type of variable and contrasted between groups by the *χ* ^2^ test for qualitative variables and the independent *t*‐test and repeated measures analysis of variance for quantitative variables. The SPSS version 22 was utilized to carry out statistical analyses with statistical significance at *p*‐value less than 0.05 (two‐sided).

## 3 RESULTS

Sixty‐four patients first enrolled in study and they were assigned to each study group with a 1:1 ratio (each group included 32 patients). Three patients were excluded from the 64 patients primarily included, due to they didn't follow‐up during study, 1 of them was from Risperidone group and 2 other were from Aripiprazole group. Thus overall, 61 patients completed the research. In the Risperidone group, the mean age of participants was 38.9 (the minimum age was 20.2 years and the maximum age was 58 years) and mean age of participants in Aripiprazole group was 40.1 (the minimum age was 21.3 years and the maximum age was 55.2 years). In terms of race, the patients in two groups were of a common Asian/Northern Iranian race. In terms of gender, in the Aripiprazole and Risperidone group in turn, 56/7% and 73/35% of participants were male, also 43/3% and 27/7% were female. In terms of weight, the average weight in Aripiperazole group was 71.3 kg and in Risperidone group was 74.8 kg.

Between the two groups, in general, no significant difference was observed in the demographic conditions and characteristics of the patients.

To comparing groups by HAM‐D score, repeated‐measures analysis of variance was used and the results shown in Table 1.

**Table 1:** Comparison between groups for HAM‐D score.

Group	Time								
	Before intervention		Six weeks after		12 weeks after		*p* Value^a^	*p* Value^b^	Effect size
	Mean	SD	Mean	SD	Mean	SD			
Aripiprazole	22.77	8.62	9.47	3.94	5.13	3.24	<0.0	0.723	0.002
Risperidone	19.6	8.19	9.53	4.52	6.87	4.11	<0.05		
*p* Value^c^	0.15		0.95		<0.10		‐		

Abbreviation: HAM‐D, Hamilton depression rating scale.

^a^ Repeated measures analysis of variance.

^b^ Group.

^c^ Independent sample *t*‐test.

As shown above, the effect of dose and timing in both Aripiprazole and Risperidone group were statistically significant (*p* = 0.002 and 0.02, respectively) and led to decrease in HAM‐D score but the effect of groups was not statistically significant and both drugs' gave the similarly reduction in HAM‐D score. Though in the 12th week of treatment, Aripiprazole was more effective in treating depression, but not statistically significant (*p* = 0.07).

To comparing groups for Y‐BOCS score, repeated‐measures analysis of variance was used and the results shown in Table 2.

**Table 2:** Comparison between groups for Y‐BOCS score.

Group	Time								
	Before intervention		Six weeks after		12 weeks after		*p* Value	*p* Value	Effect size
	Mean	SD	Mean	SD	Mean	SD			
Aripiprazole	28.1	6.96	17.17	4.66	9.37	3.36	<0.001	0.024	0.086
Risperidone	30.10	6.61	19.63	4.99	13.63	4.65	<0.001		
*p* Value	0.25		0.053		<0.001		‐		

Abbreviation: Yale−Brown obsessive‐compulsive scale.

No patient in this study experienced a worsening of OCD symptoms with either medication. As shown above, the effect of dose and timing in both Aripiprazole and Risperidone group were statistically significant (*p* < 0.001) and led to decrease in Y‐BOCS score. In addition, the effect of groups was statistically significant (*p* < 0.001), as in the 12th week of intervention, the mean Y‐BOCS score for the Aripiprazole group was significantly lower. Though in the sixth week of intervention, Aripiprazole was more effective in treating OCD but not statistically significant (*p* = 0.053).

To comparing groups for YMRS score, repeated‐measures analysis of variance was used and the results shown in Table 3.

**Table 3:** Comparison between groups for YMRS score.

Group	Time								
	Before intervention		Six weeks after		12 weeks after		*p* Value	*p* Value	Effect size
	Mean	SD	Mean	SD	Mean	SD			
Aripiprazole	12.67	8.59	2.5	2.75	0.30	0.65	<0.05	0.338	0.016
Risperidone	14.43	10.18	3.37	4.35	0.8	1.37	<0.05		
*p* Value	0.47		0.36		0.07		‐		

Abbreviation: YMRS, young mania rating scale.

As shown above, the effect of dose and timing in both Aripiprazole and Risperidone groups were statistically significant (*p* = 0.04 and 0.03, respectively) and led to decrease in the YMRS score. Still, the effect of groups was not statistically significant, though in the 12th week of treatment Aripiprazole was more effective in treating mania but not statistically significant (*p* = 0.07).

The *χ* ^2^ test was utilized to contrast adverse effects between groups, and the findings are indicated in Table 4.

**Table 4:** Adverse effects in both groups (number of persons, percentage).

Variable	Group		
	Aripiprazole	Risperidone	*p* Value
Dry mouth	0 (0)	2 (6.7)	0.49
Weight gain	1 (3.3)	16 (53.3)	<0.001
Sleep disturbance	8 (26.7)	1 (3.3)	<0.05
Nausea	4 (13.3)	0 (0)	0.11
Increased appetite	1 (3.3)	1 (3.3)	>0.99
Tremor	0 (0)	4 (13.3)	0.11
Extra‐pyramidal syndrome	0 (0)	4 (13.3)	0.11
Akathisia	0 (0)	2 (6.7)	0.49
Dizziness	1 (3.3)	1 (3.3)	>0.99
Sexual dysfunction	0 (0)	1 (3.3)	>0.99
Decrease libido	0 (0)	1 (3.3)	>0.99
Weight loss	2 (6.7)	0 (0)	0.49
Decrease appetite	1 (3.3)	0 (0)	>0.99
Increase libido	0 (0)	1 (3.3)	>0.99
Adverse effects in 6th week	9 (30)	12 (40)	0.41
Adverse effects in 12th week	12 (40)	18 (60)	0.12

As shown above, significant differences were found between the two group for weight gain and sleep disturbance in turn by (*p* < 0.001 and 0.05) as adverse effects of treatment. As shown in Table 4, Risperidone induced statistically significant weight gain (*p* < 0.001) and Aripiprazole induced statistically significant sleep disturbance (*p* < 0.05).

## 4 DISCUSSION

This present clinical trial aimed to explore the effect of adjunctive therapy of Aripiprazole and Risperidone along with valproate sodium in one of the most challenging problem in psychiatry, that is treating OCD in patients with BD and also aimed to compare their effect in treating mania and depression in these patients. We find both drug as adjunctive therapy are significantly effective in treating OCD, mania, and depression, their effectiveness is same in mania and depression, however this point is important that in 12th weeks and with dose of 20 mg/day, Aripiprazole is more effective than 4 mg/day Risperidone, in treating mania and depression but it's not analytically significant, but only in treating OCD, Aripiprazole is significantly more effective than Risperidone. So the findings of the present research indicated that adjunctive therapy of Aripiprazole and Risperidone with valproate sodium in the treatment of OCD‐BD can lead to a significant reduction in HAM‐D rating scale, Y‐BOCS, and YMRS scores which were consistent with the research findings of Albert et al.^22^ and Amerio et al.'s studies,^15^ which showed that adding Aripiprazole to the mood stabilizers could result in significant therapeutic improvement in obsessive and emotional symptoms but on the contrary of our research, they did not compare Aripiprazole and Risperidone and It is worth mentioning that their study mainly included case reports and outpatients' units, which potentially leads to selection bias, and their results could not be generalized to society. Sahraian et al.^31^ performed research to investigate the effect of Aripiprazole as adjunctive therapy for treating obsessive‐compulsive symptoms in BD. They stated that using Aripiprazole significantly reduced the mean Y‐BOCS score without any side effect. In contrast, our study showed using Aripiprazole is associated with sleep disturbance in patients, but also another difference from their research and our study is that they didn't assessed the effect of adjunctive therapy of Aripiprazole in treating depression and mania and their research did not compare between two drugs. Our study showed that adjunctive therapy with Aripiprazole and Risperidone with valproate sodium in the management of BD, significantly reduced mean YMRS score, which were consistent with the findings of Dieji et al.'s^24^ study. Due to their research, the use of the listed drugs was effective in BD type 1 or mixed type, and both Aripiprazole and Risperidone are usable. They recommend using a variety of valproate sodium and Aripiprazole in patients' who are high risk for obesity, since its less weight gain possibility, but in contrast with our study, their study included only BD not comorbidity of OCD‐BD in patients and also they didn't assessed treatment of obsessive compulsive disorder in these group of patients. However the impossibility of following‐up patients for a more extended period is the limitation of our study and evaluating over a long period of time supports the robustness of the data report. So we suggest designing similar studies with longer follow‐up period, using other anti‐psychotics drugs to compare, and also performing study to compare the CBT with antipsychotic drugs and also we recommend to perform, a cross‐over study and using the same patients as their own comparator and determining if keeping them on valproate sodium, but switching them from Aripiprazole to Risperidone or vice versa can be equally as effective.

## 5 CONCLUSION

Our results showed a significant effect of time and dosage of Aripiprazole and Risperidone as adjunctive therapy along with valproate sodium for treating OCD in BD. Both drugs similarly led to a reduction in mean HAM‐D rating scale and YMRS scores, but in treating OCD, Aripiprazole was more effective and the score of Y‐BOCS was lower significantly with Aripiprazole. In our study, weight gain due to Risperidone and sleep disturbance due to Aripiprazole were the two main significant side effects.

## AUTHOR CONTRIBUTIONS

**Faezeh Khorshidian**: Data curation; formal analysis; methodology; project administration; resources; writing—original draft; writing—review and editing. **Sakineh Javadian Koutanaei**: Conceptualization; data curation; writing—original draft; writing—review and editing. **Angela Hamidia**: Methodology; validation. **Farzan Kheirkhah**: Investigation; methodology. **Ali Akbar Moghadamnia**: Methodology. **Ali Bijani**: Formal analysis; investigation; software; validation. **Seyedeh Mahbobeh Mirtabar**: Investigation.

## CONFLICT OF INTEREST STATEMENT

The authors declare no conflict of interest.

## ETHICS STATEMENT

The initial proposal of this study was approved by the Ethics Committee of Babol University of Medical Sciences, Babol, Iran, by the code of ethics: (IRCT20200614047761N1). Before the study, the objectives of the study were fully explained to the volunteers, and informed consent was obtained from all participants of the study.

## TRANSPARENCY STATEMENT

The lead author Sakineh Javadian Koutanaei affirms that this manuscript is an honest, accurate, and transparent account of the study being reported; that no important aspects of the study have been omitted; and that any discrepancies from the study as planned (and, if relevant, registered) have been explained.

## Data Availability

The data set presented in the study is available on request from the corresponding author during submission or after publication.

## References

[1] Robbins TW , Vaghi MM , Banca P . Obsessive‐compulsive disorder: puzzles and prospects. Neuron. 2019;102(1):27‐47. 10.1016/j.neuron.2019.01.046 30946823

[2] Abramowitz JS , Jacoby RJ . Obsessive‐compulsive disorder in the DSM‐5. Clin Psychol. 2014;21(3):221‐235. 10.1111/cpsp.12076

[3] Fawcett EJ , Power H , Fawcett JM . Women are at greater risk of OCD than men: a meta‐analytic review of OCD prevalence worldwide. J Clin Psychiatr. 2020;81(4):19r13085. 10.4088/JCP.19r13085 32603559

[4] Drubach DA . Obsessive‐compulsive disorder. CONTIN Lifelong Learn Neurol. 2015;21(3):783‐788. 10.1212/01.CON.0000466666.12779.07 26039854

[5] Jaisoorya T , Janardhan Reddy Y , Nair B , et al. Prevalence and correlates of obsessive‐compulsive disorder and subthreshold obsessive‐compulsive disorder among college students in Kerala, India. Indian J Psychiatr. 2017;59(1):56‐62. 10.4103/0019-5545.204438 PMC5419013 28529361

[6] Pinto A , Eisen JL. Personality features of OCD and spectrum conditions. The Oxford Handbook of Obsessive‐Compulsive and Spectrum Disorders. Oxford Handbooks Online; 2016. 10.1093/oxfordhb/9780195376210.001.0001

[7] Karanti A , Kardell M , Joas E , Runeson B , Pålsson E , Landén M . Characteristics of bipolar I and II disorder: a study of 8766 individuals. Bipolar Disord. 2020;22(4):392‐400. 10.1111/bdi.12867 31724302

[8] Amerio A , Stubbs B , Odone A , Tonna M , Marchesi C , Nassir Ghaemi S . Bipolar I and II disorders; a systematic review and meta‐analysis on differences in comorbid obsessive‐compulsive disorder. Iran J Psychiatr Behav Sci. 2016;10(3):e3604. 10.17795/ijpbs-3604 PMC5098723 27826323

[9] Peng D , Jiang K . Comorbid bipolar disorder and obsessive‐compulsive disorder. Shanghai Arch Psychiatr. 2015;27(4):246‐248. 10.11919/j.issn.1002-0829.215009 PMC4621290 26549961

[10] Amerio A , Odone A , Liapis CC , Ghaemi SN . Diagnostic validity of comorbid bipolar disorder and obsessive–compulsive disorder: a systematic review. Acta Psychiatr Scand. 2014;129(5):343‐358. 24506190 10.1111/acps.12250

[11] Cederlöf M , Lichtenstein P , Larsson H , et al. Obsessive‐compulsive disorder, psychosis, and bipolarity: a longitudinal cohort and multigenerational family study. Schizophr Bull. 2015;41(5):1076‐1083. 10.1093/schbul/sbu169 25512596 PMC4535627

[12] Vázquez GH , Baldessarini RJ , Tondo L . Co‐occurrence of anxiety and bipolar disorders: clinical and therapeutic overview. Depress Anxiety. 2014;31(3):196‐206. 24610817 10.1002/da.22248

[13] Sharma LP , Reddy Y . Obsessive–compulsive disorder comorbid with schizophrenia and bipolar disorder. Indian J Psychiatr. 2019;61(suppl 1):140. 10.4103/psychiatry.IndianJPsychiatry_527_18 PMC6343407 30745688

[14] Amerio A . Suicide risk in comorbid bipolar disorder and obsessive‐compulsive disorder: a systematic review. Indian J Psychol Med. 2019;41(2):133‐137. 30983660 10.4103/IJPSYM.IJPSYM_367_18 PMC6436414

[15] Amerio A , Odone A , Liapis CC , Ghaemi SN . Diagnostic validity of comorbid bipolar disorder and obsessive‐compulsive disorder: a systematic review. Acta Psychiatr Scand. 2014;129(5):343‐358. 10.1111/acps.12250 24506190

[16] Lochner C , Chamberlain SR , Kidd M , et al. The effects of acute serotonin challenge on executive planning in patients with obsessive‐compulsive disorder (OCD), their first‐degree relatives, and healthy controls. Psychopharmacology. 2020;237(10):3117‐3123. 10.1007/s00213-020-05597-7 32638035 PMC7116135

[17] Mohyadini H , Bakhtiar Pour S , Pasha R , Ehteshmzadeh P . Effectiveness of cognitive‐behavioral treatment community on progress in patients with obsessive‐compulsive disorders. Women's Health Bulletin. 2021;8(1):18‐25. 10.30476/whb.2021.87708.1075

[18] de Filippis R , Aloi M , Bruni A , Gaetano R , Segura‐Garcia C , De Fazio P . Bipolar disorder and obsessive compulsive disorder: the comorbidity does not further impair the neurocognitive profile. J Affect Disord. 2018;235:1‐6. 29627704 10.1016/j.jad.2018.03.010

[19] Grande I , Vieta E . Pharmacotherapy of acute mania: monotherapy or combination therapy with mood stabilizers and antipsychotics? CNS Drugs. 2015;29(3):221‐227. 25711483 10.1007/s40263-015-0235-1

[20] Goodman WK , Storch EA , Sheth SA . Harmonizing the neurobiology and treatment of obsessive‐compulsive disorder. Am J Psychiatr. 2021;178(1):17‐29. 10.1176/appi.ajp.2020.20111601 33384007 PMC8091795

[21] Amerio A , Odone A , Ghaemi SN . Aripiprazole augmentation in treating comorbid bipolar disorder and obsessive‐compulsive disorder: a systematic review. J Affect Disord. 2019;249:15‐19. 10.1016/j.jad.2019.02.024 30743017

[22] Albert U , Marazziti D , Di Salvo G , Solia F , Rosso G , Maina G . A systematic review of evidence‐based treatment strategies for obsessive‐compulsive disorder resistant to first‐line pharmacotherapy. Curr Med Chem. 2019;25(41):5647‐5661. 10.2174/0929867325666171222163645 29278206

[23] Lai J , Lu Q , Zhang P , Xu T , Xu Y , Hu S . Aripiprazole augmentation in managing comorbid obsessive‐compulsive disorder and bipolar disorder: a case with suicidal attempts. Neuropsychiatr Dis Treat. 2017;13:87. 10.2147/NDT.S122316 28096676 PMC5207469

[24] Dieji B , Nazeri Astane A , Rezaie O , Biglarian A , Amanat N . Comparison of the efficacy of risperidone and aripiprazole in combination with sodium valproate in patients with acute manic or mixed episodes. Iranina J Psychiatr Nur. 2017;5(2):52‐59.

[25] Veale D , Miles S , Smallcombe N , Ghezai H , Goldacre B , Hodsoll J . Atypical antipsychotic augmentation in SSRI treatment refractory obsessive‐compulsive disorder: a systematic review and meta‐analysis. BMC Psychiatr. 2014;14(1):317. 10.1186/s12888-014-0317-5 PMC4262998 25432131

[26] Esfahani SR , Motaghipour Y , Kamkari K , Zahiredin A , Janbozorgi M . Reliability and validity of the Persian version of the Yale‐Brown obsessive‐compulsive scale (Y‐BOCS). IJPCP. 2012;17(4):297‐303.

[27] Daihui PE , Jiang K . Comorbid bipolar disorder and obsessive‐compulsive disorder. Shanghai Arch Psychiatr. 2015;27(4):246. 10.11919/j.issn.1002-0829.215009 PMC4621290 26549961

[28] Leucht S , Crippa A , Siafis S , Patel MX , Orsini N , Davis JM . Dose‐response meta‐analysis of antipsychotic drugs for acute schizophrenia. Am J Psychiatr. 2020;177(4):342‐353. 31838873 10.1176/appi.ajp.2019.19010034

[29] Gharaei B , Mehryar M , Mehrabi F . Attribution style in patients with anxiety and depression comorbidity. Iran J Psychiatr Clin Psychol. 2000;5(4):99.

[30] Ebrahimi A , Barekatain M , Bornamanesh A , Nassiri H . Psychometric properties of the Persian version of bipolar depression rating scale (BDRS) in patients and general population. Iran J Psychiatr Clin Psychol. 2015;21(1):60‐68.

[31] Sahraian A , Ehsaei Z , Mowla A . Aripiprazole as an adjuvant treatment for obsessive and compulsive symptoms in manic phase of bipolar disorder: a randomized, double‐blind, placebo‐controlled clinical trial. Prog Neuropsychopharmacol Biol Psychiatr. 2018;84:267‐271. 10.1016/j.pnpbp.2018.03.014 29544694

